# A script‐enabled interactive checklist document for efficient management of electronic devices in a busy multimodality radiotherapy clinic

**DOI:** 10.1002/acm2.14302

**Published:** 2024-02-18

**Authors:** Mark D. Pepin, Eric E. Brost, Kristi A. Klein, Yolanda I. Garces, Debra H. Brinkmann

**Affiliations:** ^1^ Department of Radiation Oncology Mayo Clinic Rochester Minnesota USA

**Keywords:** implanted electronic medical devices, information resources, process of care, quality management, scripting

## Abstract

**Purpose:**

Develop an efficient, interactive, and instructive checklist document for the management of implanted electronic medical devices in a multimodality radiotherapy clinic.

**Methods:**

The built‐in scripting and interactivity of a popular commercial word processor was used to develop an interactive document that changes the information presented to the user based on drop‐down selections. The interactivity and scripting were compatible with the radiation oncology information system (ROIS) which allows the document to be accessible by all team members and serve as a permanent record in a patient's electronic chart.

**Results:**

The final interactive document, which was clinically deployed after beta testing with a group consisting of nurses and medical physicists, presents information and action plans to the user based on multiple departmental medical device decision trees that are specific to the combination of device, treatment modality, rhythm‐pacing dependence for cardiac devices, and distance from the device to the treatment volume.

**Conclusion:**

A script‐enabled interactive document was developed for a busy multimodality clinic, condensing multiple comprehensive departmental guidelines spanning multiple device types and treatment modalities into a single interactive checklist accessible within the ROIS. Given the wide accessibility of the commercial word processor, this approach could be adopted by other clinics to streamline their own respective workflows.

## 1 INTRODUCTION

Patients undergoing radiotherapy can present with a wide variety of implanted electronic devices that require the radiation oncology care team to assess and, where prudent, implement additional patient‐safety measures due to the potentially harmful effects of radiation on the devices.^1^, ^2^ These effects have been detailed in the report of the AAPM's Task‐Group 203^1^ (TG‐203) in the context of cardiac implanted electronic devices (CIEDs) and include device malfunction from cumulative dose, dose rate, and neutron‐induced single‐event upset (SEUs). The impact from such effects has led to several consensus guidelines including TG‐203^1^ and others^3^, ^4^ for CIEDs. The same radiation effects can also affect non‐cardiac devices, though the result of a device failure will vary by the device's function leading to additional guidelines for non‐cardiac devices.^2^, ^5^

The relative risk of potential failure modes for an irradiated device differs based on the modality of treatment being received. Our clinic treats with pencil‐beam scanning (PBS) proton therapy as well as linac‐based x‐ray therapy. In x‐ray therapy, the out of field dose received by a device is primarily from scattered photons while in proton therapy it is from secondary neutrons.^6^ Although the measured neutron dose for PBS is less than both passively‐scattered proton therapy and 18 MV x‐ray therapy,^1^, ^6^, ^7^ a SEU could potentially lead to catastrophic failure, in particular for CIEDs, leading to enhanced treatment guidelines in PBS proton therapy.^1^, ^8^, ^9^ The workflow and experience in our clinic for treating CIED patients with PBS protons has been previously reported.^10^

This study's purpose was to design a single clinical checklist document that was flexible to accommodate differing guidelines based on device type and treatment modality, while efficiently streamlining the quick workflow of our busy clinic. This was accomplished using scripting functionality compatible with our clinical radiation oncology information system (ROIS).

## 2 METHODS

### 2.1 Operating in a multimodality clinic

The external beam therapy modalities available at our clinic consist of 12 Varian C‐arm linear accelerators, two Varian Ethos linear accelerators, and a Hitachi Probeat V synchrotron system feeding to four PBS treatment gantries. Eight of the C‐arm accelerators are at regional satellite facilities, while the rest are at our central campus. For the remainder of this manuscript, a reference to the “x‐ray clinic” includes both the main campus and all satellite facilities which follow the same workflow. In 2022, we treated > 5500 and > 1200 courses of radiation in our x‐ray and proton clinics, respectively, with an average time from simulation to treatment of 3.5 days for the central x‐ray clinic.

Our department has implemented multiple internal guideline documents for management of radiotherapy patients with implanted electronic medical devices. Separate decision trees were developed in collaboration with the Cardiology department for patients with cardiac devices being treated with proton therapy due to the higher potential for SEUs compared to ≤10 MV x‐rays. Guidelines for non‐cardiac devices treated with either modality were determined in collaboration with the respective multidisciplinary specialty clinics.

### 2.2 General workflow and document sections

**FIGURE 1:**
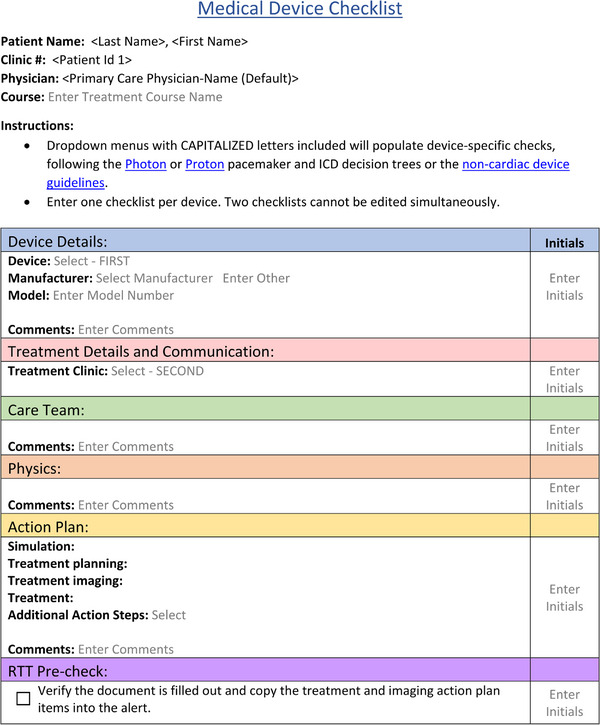
The initial blank Medical Device Checklist and Action Plan document. The user makes selections from dropdowns to indicate the type of device and the treatment clinic (x rays or protons) after which device‐ and modality‐specific recommendations appear.

When a new patient enters our clinic's workflow they are asked at several junctions, including during the initial consultation with the treating radiation oncologist, whether they have any implanted medical devices. If the answer is yes, a task is given to the nursing team to determine device type and model and begin the triage process to determine if further action is required. Part of this process is importing a Medical Device Action Plan and Checklist template in Aria v15.6 (Varian, Palo Alto, California, USA), our clinic's ROIS. This document is used for each course of treatment, and it interactively guides the radiation‐oncology team on what actions are needed. The template starts with no selectable fields entered as in Figure 1 and gradually populates tasks for the nursing care team, the medical physics team, and the final action plan based on selections made by the user. The document's action plan is split into four time points of the radiotherapy process which may require a specific action, typically taken by the radiation therapy technologist (RTT) or dosimetry teams. These time points are at initial CT simulation, treatment planning, image guidance used for daily patient alignment, and treatment itself. Additionally, it prompts the care team (which includes physicians, advanced practitioners, and/or registered nurses) to schedule any appropriate appointments in other specialty clinics (e.g., cardiology for CIEDs) and on‐treatment monitoring.

Based on the device type and treatment modality, initially selected by the nursing staff, a medical physicist then reviews the document to verify if any steps beyond the standard for the device and modality combination are necessary for the CT simulation and treatment planning action plans. The Action Plan fields are pre‐populated given the interactive nature of the document; however, the medical physicist and other care team members can also enter free text in a comments section for unique cases. After contouring and treatment planning is completed, a medical physicist again interacts with the document to confirm that the treatment planning action plan was followed and to determine if a special medical physics consult is required based on the device type, treatment modality, and the distance from the device to the treatment target. If the device was within the CT scan range, the device would be contoured and the distance from target to device determined by an ESAPI script (independent from the scripts within the checklist document) that calculated the closest distance from the device contour to the 50% isodose line. If the device was not within the CT scan range, the medical physicist would use CT scout images for estimation; including the device in the scouts is one of the pre‐populated action plan items at CT simulation.

The RTTs perform a final check of the patient chart before treatment begins. During this process, they confirm that all sections of the document have been completed (reaching out to the appropriate team member if not) and copy any treatment and treatment imaging action plan items into a patient alert that displays each day at treatment.

### 2.3 Document scripting

The checklist document was created using Microsoft Word (Microsoft, Redmond, Washington, USA) with the interactivity governed by macros written in Microsoft's Visual Basic for Applications (VBA) scripting language. Crucially, Aria, by default, allows macro‐enabled Word documents in its Documents workspace which permits all members of the department to interact with one centrally stored and accessible document. Note it is possible to disable macros in Aria 15.6 and instructions for individual clinics to check these settings are included in the Supplementary Material.

Microsoft Word provides built in “Content Controls” in the form of check boxes, drop‐down menus, and free‐text fields. Using VBA, macros can be tasked to run when the user enters or exits a content control. Sections of text can be assigned as a “Bookmark” which can be accessed as objects by VBA. The macros read new values from a drop‐down content control and manipulate parts of the document accordingly. Specifically, all potential options for any device were pre‐written, assigned as individual bookmarks, and then set as hidden text. When a specific device and treatment modality are selected, bookmarks relevant to those selections are unhidden by the macros and presented to the user for further action. The interoperability of Aria with interactive Word features additionally extends to bookmarks; if specifically named bookmarks are used, Aria auto‐populates demographic information such as the patient's name, medical record number and the treating physician. The document was developed and tested outside of Aria with new versions uploaded through the Aria Data Administration application; access and rights to Data Administration was required.

### 2.4 Interactive branching

**FIGURE 2:**
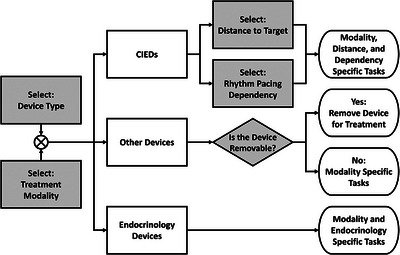
Flow diagram demonstrating left‐to‐right the main branching points in the document scripting. Different branches lead to differing options presented to the user and different action plans to be taken for device management. Shaded steps indicate that user interaction is required. CIED, cardiac implanted electronic device.

While many of the presented options are simple check boxes to indicate a certain task was performed, there are specific drop‐down selectors whose content greatly changes what is presented to the user. These main branching points are shown by the diagram in Figure 2. The type of device and treatment clinic are the two primary decisions; the document workflow only begins once both are selected. Only the main branching points are illustrated; some non‐CIED devices also require a distance to determine whether a consultation in the specialty clinic is warranted. The types of devices were sorted into three main categories as seen in Table 1.

**TABLE 1:** General categorization of device types implemented in our checklist.

Cardiac implanted electronic devices	Endocrinology devices	Other devices
Pacemaker Cardioverter defibrillator Ventricular assist device Loop recorder/cardiac monitor	Traditional Insulin pump Tubeless insulin pump Continuous glucose monitor	Neurostimulator Sacral neuromodulator Chemotherapy drug infusion pump Anesthesia drug infusion pump Pulmonary hypertension infusion pump Cochlear Implant Ventriculoperitoneal Shunt Other (electronic or not)

If a CIED device is selected, the decision trees diverge rapidly depending on the treatment clinic, how dependent the patient is on the device, and, for the x‐ray clinic, how close the device is to the treatment region. In contrast, the on‐treatment management of endocrinology devices, such as insulin pumps and monitors, is standardized across the treatment modalities. The “other devices” categories span various neurostimulators, infusion pumps, cochlear implants, ventriculoperitoneal shunts, and other non‐specified devices that can be present. Some of these devices could be removed for treatment, resulting in no action plan needed beyond removal, while others have device specific action items, e.g., having a spare pulmonary infusion pump on hand in case of failure.

## 3 RESULTS

**FIGURE 3:**
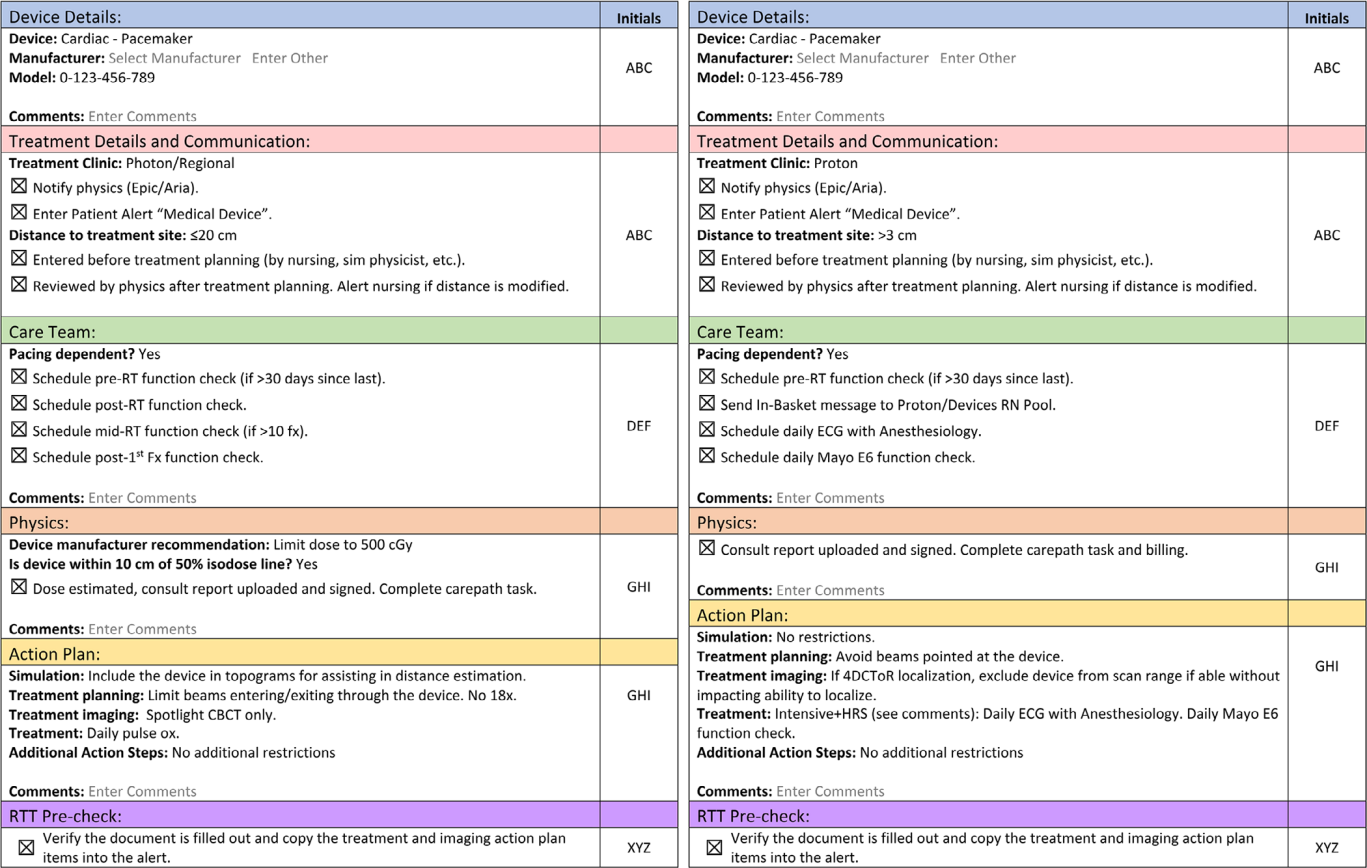
Representative checklist documents for treatment in the x‐ray (left) and proton (right) clinics populated for a patient that is dependent on a pacemaker. All text was auto generated based on the user's selections.

Two examples of final checklist documents for CIEDs are given in Figure 3, showing divergent action plans based on treatment with protons as compared with x rays. The text and options shown were auto populated based on the user's selections in the “Device,” “Treatment Clinic,” “Distance to treatment site,” “Pacing Dependent?”, and “Is device within 10 cm of 50% isodose line” options. The figure highlights the different decision trees between the two treatment modalities including differences in planning technique (restriction on beam energy for the x‐ray clinic to ≤10 MV to avoid neutron production), on‐treatment monitoring (pulse oximetry vs. daily ECG for the x‐ray and proton clinics, respectively), frequency of device function checks (single mid‐treatment vs. daily for the x‐ray and proton clinics, respectively), and daily image guidance (due to the different volumetric imaging technology available; CBCT and CT‐on‐rails for the x‐ray and proton clinics, respectively). These are generic examples; guidelines and dose limits may vary based on specific manufacturer recommendations.

**FIGURE 4:**
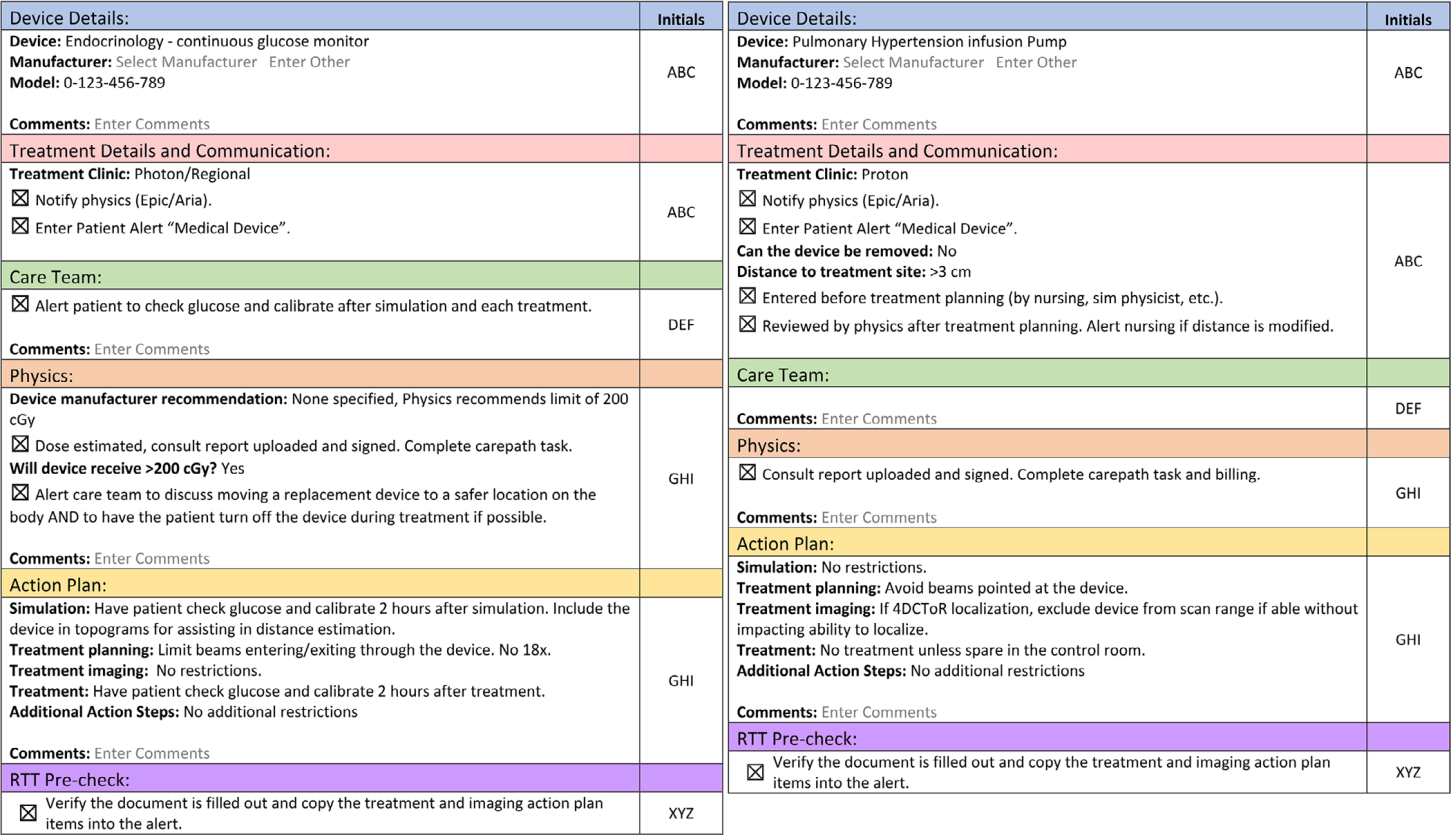
Representative checklist documents for a patient with a continuous glucose monitor treated in the x‐ray clinic (left) and a patient with a pulmonary hypertension infusion pump treated in the proton clinic (right).

Figure 4 demonstrates two more representative checklist documents that were filled out for an endocrinology device treated in the x‐ray clinic and a pulmonary hypertension infusion pump treated in the proton clinic. These demonstrate how auto populated fields differ for the two non‐CIED branches in Figure 2.

## 4 DISCUSSION

The multiple guideline documents used by our clinic are in‐depth, detailed, and subsequently long documents to parse. Consulting such detailed guidance can be prone to errors or omissions and AAPM Medical Physics Practice Guideline 4.a recommends a checklist as an approach to mitigate such risk.^11^ As such, one of the goals of the interactive checklist was to reduce the frequency at which individual team members would need to consult the full‐length guidance documents. This is especially important since at times it may be weeks between when an individual nurse or medical physicist has a case with a specific type of device. As an automated process is less error prone than manual recall, after the new document's release, some team members were presented with management options for some devices they had not been considering before. In some cases, this led to practice quality improvement efforts to either update the guidelines to remove outdated recommendations or re‐emphasize guidelines that were misremembered.

Releasing the interactive document to the full clinic required forethought to training on its use. A peculiarity of how Microsoft Word handles its content controls is that macros only run after the user fully exits the field, either by hitting the “Tab” key or clicking elsewhere in the document. This led to confusion among some of the early testers, when no options were presented, requiring additional training for those who interact with the document. This was accomplished by training a handful of individuals in each work group who then became point people for the rest of their team. Another important consideration in the document's VBA programming was that multiple team members would interact with it at separate times and variables defined and used by the macros could be cleared between uses in Aria. The state of the document thus needed to be checked upon entry of a content control field, and all variables strictly re‐assigned, to ensure the correct script was executed upon exit.

One limitation of using scripting in such documents is the linear directionality of the workflow. The further down into the workflow past branching points that a user goes, the more challenging it is from a scripting perspective to accurately change the options if, e.g., the user goes back to the beginning and changes the treatment modality. One such near miss occurred with an early version of the document for a CIED patient who initially was to receive x‐ray treatment but then switched to a proton treatment and the required on‐treatment monitoring was not scheduled until the morning before treatment began. To prevent future occurrences, a safety net was built into the macros such that if the user changes either the device type or treatment modality the document and its fields are reset, forcing the user to start from a fresh checklist. A pop‐up is presented to the user to confirm that they want this to occur. Alternatively, the document can be “errored out” in Aria (hidden by default, but still present in the patient's record) and a new one inserted if there is any uncertainty about making changes later in the pre‐treatment process. Building more “backwards compatibility” into the document is an area for future improvements.

The checklist document was constructed to follow the guidance recommendations for a single device type. It is not uncommon, however, for patients to present with more than one device. Our standard procedure, as indicated in the header material in Figure 1, was to insert and fill out a separate document for each device. One exception to this process was allowed for the common combination of a patient having both an insulin pump and a continuous glucose monitor. The action steps for these devices did not conflict and thus a combined option in the device selection list was given such that options for both devices are presented. Adding more common device combinations as guidance becomes available is an area of ongoing improvement.

Another area for future development is to automatically populate the dose limit for the device based on the selected manufacturer if an individual manufacturer has a standard recommendation. If this were to be implemented, reviewing the standard recommendation at some frequency would still be recommended to avoid disconnect if that recommendation were to change in the future. At the release of the checklist document, the medical physicist was responsible for identifying and selecting the dose limit. If none was provided by the manufacturer, our department's standard policy was to limit dose to < 200 cGy (as demonstrated in the left panel of Figure 4).

Two medical physicists jointly developed the checklist document and the associated macros. To mitigate the likelihood of software bugs reaching the clinically deployed version, when one physicist made a change the other then performed a quality‐control test. The document was also developed using Microsoft's OneDrive for Business application which included built in version‐control functionality. Furthermore, given the potentially divergent nature of recommendations between the x‐ray and proton clinics, one medical physicist from each clinic is involved to stay current with recommendations. This duplicate‐development structure and ongoing internal documentation also made the document's future viability more robust to staff turnover.

## 5 CONCLUSION

An interactive multimodality checklist document was created and clinically deployed for the management of patients with implanted medical devices in a large high‐volume radiotherapy clinic. The document condensed the requirements from multiple comprehensive guidelines, including for CIEDs treated with either x‐ray or proton radiotherapy, into a single location to reduce the amount of time needed by staff to consult and parse the in‐depth documents. The interactivity was accomplished by leveraging the built‐in functionality of Microsoft Word and its VBA scripting environment, something that is accessible to all institutions who use the common document‐creation software. This approach could thus be adopted by other clinics to streamline their own workflow and reduce the burden of treating patients with implanted medical devices.

## AUTHOR CONTRIBUTIONS

Conception of the document: Eric Brost. Design and software development for the document: Mark Pepin, Eric Brost. Conception and design for the device guidelines: Yolanda Garces, Debra Brinkmann, Kristi Klein. Draft manuscript preparation: Mark Pepin, Eric Brost. All authors reviewed and approved the final version of the manuscript.

## CONFLICT OF INTEREST STATEMENT

The authors have no relevant conflicts of interest to disclose.

## Supporting information




Supporting Information

## Data Availability

Data sharing not applicable to this article, as no datasets were generated or analyzed during the current study.
