# Supplementary material for: A script‐enabled interactive checklist document for efficient management of electronic devices in a busy multimodality radiotherapy clinic
Source: J Appl Clin Med Phys. 2024 Feb 18;25(3):e14302. doi: 10.1002/acm2.14302 (PMC10929987; doi:10.1002/acm2.14302)
Supplement: Supplementary file 1 — Supporting Information [file ACM2-25-e14302-s001.pdf]

SUPPLEMENTAL MATERIAL: EXAMPLE VBA CODE FOR UNHIDING/HIDING BOOKMARKED TEXT

Included in this supplement is an example of VBA code that can be used to unhide or hide text assigned as a bookmark in a Word document. The prerequisites for this code to function are as follows: the Developer tab must be enabled, two lines of text must be highlighted and assigned with two different bookmarks ("bm1" and "bm2" in the code below), a "Drop-Down List Content Control" must be inserted to execute the subroutine (assigned with the title and tag "dd1" in the code below) and given the options: "Select", "Show bm1", "Show bm2", "Hide all". The drop-down properties do not need to be linked to the VBA code, it will execute the subroutine upon exiting the field, through hitting "Tab" key or clicking elsewhere. Within Aria's Data Administration software, under the Clinical Assessment → Patient Documents → General tab, the following two options must be unchecked: "Disable Microsoft Word Add-ins", "Disable Word Developer Tab". Of note, Aria v15.6 cannot import Word documents that have more than two "_" characters within the bookmark name, though bookmarks inserted through Aria's Data Administration software will have more than two of these characters, i.e., the bookmark for automatic population of the patient ID will have the bookmark name "T0003_343_0_0_0_00000". Bookmarks can be successfully nested within one another.

```
Option Explicit ‘ assign global variable names, allowing them to be reused
without needing redefining in new subroutines
Global bm1 As Range
Global bm2 As Range
Global dd1 As ContentControl

Sub DefineVariables() ‘ this subroutine can be executed within other
subroutines to ensure that the document can be modified by multiple users at
different time points
Set bm1 = ActiveDocument.Bookmarks("bm1").Range
Set bm2 = ActiveDocument.Bookmarks("bm2").Range
Set dd1 = ActiveDocument.SelectContentControlsByTitle("dd1").Item(1)

Sub ResetBookmarks() ‘ this subroutine can be executed within other
subroutines to ensure that the document will always show the correct result
```

if a drop-down menu is changed multiple times. If multiple drop-down menus are in the document, each one will need its own reset subroutine.

```
bm1.Font.Hidden = True
bm2.Font.Hidden = True

Private Sub Document_ContentControlOnExit (ByVal cc As ContentControl, Cancel
As Boolean) ‘this is the workhorse subroutine that executes the show/hide
code and has been generalized for multiple content control drop-downs. This
code must go under the “ThisDocument” section of the VBA development window.
DefineVariables

Application.ScreenUpdating = False ‘this line helps speed up subroutine
operation in Aria
Select Case cc.Title
Case "dd1"
Select Case dd1.Range.Text
      Case "Select" ‘ if Select, do nothing
      Case "Show bm1"
            ResetBookmarks
            bm1.Font.Hidden = False
      Case "Show bm2"
            ResetBookmarks
            bm2.Font.Hidden = False
      Case "Show bm2"
            ResetBookmarks
      End Select
End Select
Application.ScreenUpdating = True ‘show the result of the operation
```
